# Adaptive teaching, self-regulated learning and mathematics learning outcomes: a social cognitive model of learner agency in vocational education

**DOI:** 10.3389/fpsyg.2026.1921285

**Published:** 2026-08-31

**Authors:** Shiqin Peng, Tongyao Han

**Affiliations:** 1 Basic Teaching Department, Yunnan Vocational College of Mechanical and Electrical Technology, Kunming, Yunnan, China; 2 Faculty of Human Development, Universiti Pendidikan Sultan Idris, Proton City, Tanjong Malim, Perak, Malaysia

**Keywords:** adaptive teaching, learner agency, mathematics learning outcomes, self-regulated learning, vocational mathematics education

## Abstract

**Introduction:**

This study examined the associations among adaptive teaching practices, self-regulated learning (SRL), and mathematics learning outcomes in vocational education, with SRL conceptualized as a form of learner agency within Zimmerman's social-cognitive model of self-regulated learning.

**Methods:**

A quantitative cross-sectional design was used with questionnaire data from 350 vocational education students enrolled in mathematics-related courses. Confirmatory factor analysis and structural equation modeling were conducted using SPSS and AMOS. The indirect association through SRL was assessed using 2,000 bootstrap resamples.

**Results:**

Adaptive teaching was positively associated with SRL (β= 0.520, *p* < 0.001) and mathematics learning outcomes (β= 0.125, *p* < 0.001). SRL was positively associated with mathematics learning outcomes (β= 0.241, *p* < 0.001). The standardized indirect association between adaptive teaching and mathematics learning outcomes through SRL was 0.125, supporting partial statistical mediation.

**Discussion:**

The findings indicate that adaptive instructional support and learner self-regulation are complementary components of vocational mathematics learning. The results extend SRL theory by representing teacher-provided adaptive support and learner regulation within a single structural model. Because the study used a cross-sectional design, the indirect association should not be interpreted as causal evidence.

## 1 Introduction

Mathematics is central to vocational education because technical reasoning, measurement, quantitative problem solving and workplace decision-making frequently depend on mathematical knowledge. Vocational classrooms are also characterized by substantial variation in prior preparation, occupational orientation and confidence in mathematics. Such heterogeneity makes uniform instruction difficult to sustain and provides a strong rationale for differentiated and adaptive forms of teaching (Tomlinson, 2014; Deunk et al., 2018).

Adaptive teaching refers to deliberate adjustments in instructional strategies, task difficulty, pacing, feedback and assessment in response to students' needs and learning progress. In mathematics-related vocational courses, these adjustments can provide targeted scaffolding, address misconceptions and connect abstract concepts with occupationally relevant applications. Recent work on adaptive learning likewise emphasizes personalized support, feedback and flexible learning pathways as mechanisms through which instructional environments can respond to learner differences (Rincón-Flores et al., 2024). Persistent misconceptions and problem-solving errors can hinder students' mathematics learning, further emphasizing the importance of instructional support that responds to differences in students' prior understanding (Booth et al., 2014).

Instructional adaptation, however, does not by itself explain how students manage the learning opportunities that teachers provide. Self-regulated learning (SRL) concerns the processes through which learners set goals, select strategies, monitor performance, regulate effort and reflect on outcomes (Zimmerman, 2002; Panadero, 2017). These processes are particularly important in vocational education, where students are expected to apply knowledge independently and to move between classroom, practical and workplace-relevant tasks. Research in vocational settings has shown that planning, monitoring, feedback use, motivation and social support are important elements of students' self-regulatory development (Pylväs et al., 2022; Mejeh and Held, 2022; Sirk, 2025).

Zimmerman's model provides a useful lens for explaining the connection between adaptive teaching and learner agency. The model describes SRL as a cyclical process involving forethought, performance and self-reflection. External scaffolds can support these phases by clarifying goals, adjusting challenge, providing feedback and helping learners monitor progress. Recent research on the distribution of regulation between learning environments and learners similarly indicates that scaffolding can shape who regulates learning and how regulation is enacted (Herold and Seufert, 2025).

Four gaps motivate the present study. Theoretically, adaptive teaching and SRL are often discussed as separate instructional and learner-level constructs, leaving the pathway between environmental support and learner agency insufficiently specified. Practically, vocational mathematics teachers need evidence about how instructional adaptation can be combined with strategies that strengthen students' capacity to regulate learning. Empirically, comparatively little research has examined adaptive teaching, SRL and mathematics outcomes simultaneously within vocational education. Methodologically, an integrated latent-variable model can distinguish the measurement of the constructs from the structural associations among them and can estimate both direct and indirect associations while accounting for measurement error.

Accordingly, this study examines adaptive teaching as an environmental scaffold, SRL as the principal learner-agency mechanism, and mathematics learning outcomes as the educational outcome. Its epistemological contribution is to treat the proposed relationships as probabilistic associations that can be tested and potentially falsified rather than as deterministic causal claims. Its applied contribution is to identify whether vocational mathematics instruction may be more effective when teacher adaptation and learner self-regulation are considered together.

### 1.1 Research objectives and hypotheses

The study has four objectives: (1) to examine the association between adaptive teaching practices and students' SRL; (2) to examine the association between adaptive teaching practices and mathematics learning outcomes; (3) to examine the association between SRL and mathematics learning outcomes; and (4) to determine whether SRL statistically mediates the association between adaptive teaching practices and mathematics learning outcomes.

**H1:** Adaptive teaching practices are positively associated with students' self-regulated learning.
**H2:** Adaptive teaching practices are positively associated with students' mathematics learning outcomes.
**H3:** Self-regulated learning is positively associated with students' mathematics learning outcomes.
**H4:** Self-regulated learning statistically mediates the association between adaptive teaching practices and students' mathematics learning outcomes.

## 2 Literature review

### 2.1 Zimmerman's self-regulated learning theory and the broader social cognitive perspective

The primary theoretical framework for this study is Zimmerman's social-cognitive model of self-regulated learning. SRL is conceptualized as an active and cyclical process in which learners anticipate and plan learning, regulate cognition and behavior during task performance, and evaluate outcomes in order to adjust subsequent action (Zimmerman, 2002; Zimmerman and Schunk, 2011). This framework is particularly appropriate for vocational mathematics because learners must coordinate strategy use, persistence, time management and help seeking while applying mathematical knowledge to practical tasks.

Bandura's social cognitive theory provides the broader theoretical foundation for this model. From this perspective, learning emerges from reciprocal relations among personal factors, behavior and environmental conditions (Bandura, 1997). Adaptive teaching is therefore conceptualized as an environmental scaffold, whereas SRL represents students' agentic regulation of learning. The theoretical proposition is not that teaching mechanically produces achievement, but that instructional support may be associated with learning outcomes partly through the ways students plan, monitor and regulate their own learning.

### 2.2 Adaptive teaching practices

Adaptive teaching comprises teacher-led adjustments designed to align learning opportunities with differences in students' readiness, progress and learning needs. It can include differentiated tasks, flexible pacing, formative assessment, responsive feedback and multiple routes to the same learning goal (Tomlinson, 2014; Deunk et al., 2018). Recent adaptive-learning research also emphasizes responsiveness to learner information and feedback as a means of improving the teaching-learning process (Rincón-Flores et al., 2024).

In vocational mathematics, adaptation is especially relevant because students may enter with uneven mathematical foundations while also needing to understand how mathematical ideas transfer to technical and occupational contexts. Adaptive teaching can therefore be interpreted as both instructional differentiation and a scaffold that structures opportunities for learner regulation.

### 2.3 Self-regulated learning in vocational education

SRL includes metacognitive, motivational and behavioral processes such as goal setting, monitoring, strategy selection, effort regulation, time and study-environment management, and help seeking (Pintrich, 2004; Zimmerman, 2002). In the present study, SRL is modeled as a second-order construct comprising metacognitive self-regulation, effort regulation, time and study environment management, and help seeking.

Vocational education places distinctive demands on regulation because learning frequently alternates between structured instruction and more independent application. Pylväs et al. (2022) found that vocational students' accounts of work-based learning included goal setting, strategic planning, monitoring, social support and feedback. Mejeh and Held (2022) likewise demonstrated the relevance of explicitly promoting SRL in vocational schools. These findings support the view that SRL is not only an individual characteristic but also a capacity that can be shaped by learning conditions. The importance of responsive instructional support and the explicit development of learner self-regulation is also consistent with broader research on educational innovation, visible learning, and interventions designed to foster self-regulated learning (OECD, 2019; Perry et al., 2002; Dignath and Büttner, 2008; Hattie, 2009).

### 2.4 Adaptive teaching and self-regulated learning

Adaptive teaching may support SRL by clarifying goals, calibrating task difficulty, providing timely feedback and offering scaffolds that make monitoring and strategy adjustment easier. Such support can strengthen students‘ opportunities to engage in the forethought, performance and self-reflection phases identified by Zimmerman. At the same time, excessive external regulation could reduce learner control; therefore, the theoretically relevant question is whether adaptive teaching is positively associated with students' own regulatory capacity. Herold and Seufert (2025) highlight this distribution-of-regulation problem by showing that scaffolding and learner regulation are interdependent rather than interchangeable. This reasoning supports H1.

### 2.5 Adaptive teaching, self-regulated learning and mathematics outcomes

Adaptive teaching can be associated with mathematics outcomes through clearer explanations, more appropriate task challenge, differentiated support and stronger connections between abstract content and vocational application. Meta-analytic evidence on differentiated instruction indicates that instructional adaptation can improve educational outcomes when it is implemented systematically (Deunk et al., 2018). This provides the basis for H2.

SRL is also theoretically linked to mathematics outcomes because mathematical learning requires sustained monitoring, strategy selection, persistence and error correction. Learners who manage time and effort, evaluate understanding and seek help when necessary are better positioned to persist through complex mathematical tasks (Panadero, 2017; Zimmerman and Schunk, 2011). This reasoning supports H3.

### 2.6 The indirect role of self-regulated learning

Integrating the two perspectives suggests an indirect pathway: adaptive teaching may create conditions that support students' regulatory activity, and stronger SRL may in turn be associated with better mathematics outcomes. The expected mediation is therefore statistical and theory-informed rather than causal, because the data are cross-sectional. Testing this indirect association extends a simple input-output model of instruction by explicitly representing learner agency between instructional support and outcomes. This reasoning supports H4.

## 3 Methodology

### 3.1 Research design and philosophical orientation

The study adopted a quantitative cross-sectional design within a post-positivist orientation. The constructs were treated as fallible empirical representations of theoretically defined concepts, and the hypotheses were evaluated as probabilistic associations rather than deterministic causal relations (Creswell, 2014). Structural equation modeling (SEM) was selected because the study involved multiple latent constructs and required simultaneous evaluation of the measurement model and the hypothesized structural relations. SEM also permits explicit treatment of measurement error and estimation of direct and indirect associations within one integrated model (Kline, 2016).

### 3.2 Population, sampling and sample-size adequacy

The target population comprised vocational education students enrolled in mathematics-related courses. A stratified random sampling approach was used to obtain students from different vocational majors: students were classified by field of study and then sampled from the respective groups. The final analytic sample contained 350 valid responses.

Inclusion criteria were enrolment in the participating vocational education setting, current participation in a mathematics-related course, voluntary participation, and a questionnaire sufficiently complete for analysis. Responses outside the target population or unusable after the study's routine completeness and validity checks were excluded. The archived study documentation does not preserve a separate count for each exclusion category; therefore, no retrospective exclusion numbers are introduced in this revision.

An *a priori* power analysis was not documented in the original study records, and the revision does not retrospectively present one as prospective. To assess adequacy transparently, an RMSEA-based power calculation was conducted following MacCallum et al. (1996) using the noncentral chi-square formulation λ = (*N*- 1) dfε^2^. For the measurement model (df = 858), with *N* = 350, α = 0.05, a close-fit null *RMSEA* of 0.05 and an alternative *RMSEA* of 0.06, estimated power was approximately 0.998. This *post hoc* calculation indicates that the obtained sample was adequate for detecting this level of departure from close fit, while not replacing the value of a prospectively planned power analysis.

### 3.3 Research instruments

#### 3.3.1 Adaptive teaching practices

Adaptive teaching practices were assessed with 21 five-point Likert items. The available study documentation indicates that the item set was informed by the differentiated and adaptive instruction literature rather than reproduced from a single proprietary scale. The revised manuscript therefore describes the measure as a study-specific, literature-informed instrument and does not attribute it to an unidentified scale developer. Its content reflected differentiated tasks, pacing, feedback, assessment and instructional adjustment (Tomlinson, 2014; Deunk et al., 2018; Rincón-Flores et al., 2024). Content relevance was reviewed by educators with experience in vocational education and mathematics before use.

#### 3.3.2 Self-regulated learning

SRL items were adapted from the Motivated Strategies for Learning Questionnaire (MSLQ), originally developed and validated by Pintrich et al. (1993), with conceptual guidance from Pintrich (2004). Nineteen items represented four first-order dimensions: metacognitive self-regulation (five items), effort regulation (five items), time and study environment management (five items), and help seeking (four items). These dimensions were specified as indicators of a second-order SRL construct. The wording was adapted to the vocational mathematics context while preserving the intended regulatory dimensions.

#### 3.3.3 Mathematics learning outcomes and controls

Mathematics learning outcomes were represented by an observed mathematics outcome score based on students' self-reported performance and course-related assessment information available to the study. Prior mathematics basis and academic major were included as observed control variables because both may be associated with mathematics outcomes. Because the mathematics outcome was entered as an observed score rather than a reflective multi-item latent scale, composite reliability was not calculated for that variable.

### 3.4 Validity and reliability assessment

Content validity was addressed through expert review by educators in vocational education and mathematics. Construct validity was examined with confirmatory factor analysis (*CFA*). Standardized loadings were evaluated alongside global fit indices. Internal consistency of the latent measures was assessed using composite reliability (*CR*), calculated from the archived standardized *CFA* loadings as *CR* = (Σλ)^2^ / [(Σλ)^2^ + Σ(1 - λ^2^)]. Values around or above 0.70 were treated as evidence of acceptable composite reliability (Hair et al., 2019).

### 3.5 Data collection and ethical considerations

Questionnaires were completed during class time with the assistance of course instructors. Participants were informed of the purpose of the study, participation was voluntary, and they were informed that they could withdraw. Anonymity and confidentiality were maintained and no directly identifying information was collected. Completed questionnaires were checked for completeness and validity before analysis. Formal approval from an independent ethics committee was not obtained for the study; no retrospective ethics approval or approval number is claimed in this manuscript.

### 3.6 Data analysis

Data were analyzed using IBM SPSS and AMOS (IBM Corp., Armonk, NY, United States). Descriptive statistics and Pearson correlations were examined first. CFA was then used to assess the measurement model, followed by SEM to evaluate the hypothesized associations among adaptive teaching, SRL and mathematics learning outcomes while including mathematics basis and academic major as controls. Model fit was evaluated using chi-square divided by degrees of freedom (χ^2^/df), comparative fit index (*CFI*), Tucker-Lewis index (*TLI*), incremental fit index (*IFI*) and root mean square error of approximation (*RMSEA*), with interpretation informed by Hu and Bentler (1999) and Kline (2016). A bootstrap procedure with 2,000 resamples was used to assess the indirect association through SRL. Because the study is cross-sectional, mediation is interpreted as a statistical indirect association and not as evidence of temporal or causal mediation (Preacher and Hayes, 2008).

## 4 Results

### 4.1 Descriptive statistics, correlations and composite reliability

Table 1 presents the descriptive statistics, correlations and composite reliability information available from the CFA. Adaptive teaching and SRL showed moderate average levels, while mathematics learning outcomes showed a slightly higher mean and greater variability. Adaptive teaching correlated positively with SRL and mathematics learning outcomes, and SRL correlated positively with mathematics learning outcomes. Composite reliability was 0.927 for adaptive teaching and 0.849 for the second-order SRL construct.

**Table 1:** Descriptive statistics, correlations and composite reliability.

Variable	M	SD	CR	1	2	3
1. Adaptive teaching practices	3.00	0.77	0.927	-		
2. Self-regulated learning	3.00	0.77	0.849	0.373^**^	-	
3. Mathematics learning outcomes	3.12	1.51	n/a	0.363^**^	0.423^**^	-

CR = composite reliability. CR is not applicable to the observed mathematics outcome score. ^**^p < 0.01.

### 4.2 Measurement model evaluation

CFA indicated an acceptable but not optimal measurement-model fit: χ^2^(858) = 1365.07, χ^2^/df = 1.59, *CFI* = 0.89, *TLI* = 0.89, *IFI* = 0.89 and *RMSEA* = 0.041, 90% *CI* [0.037, 0.045]. The χ^2^/df and RMSEA supported satisfactory absolute fit, whereas the incremental fit indices were marginally below the conventional.90 reference point. The measurement model was therefore retained for structural analysis with explicit caution regarding marginal incremental fit. The measurement-model fit indices are summarized in Table 2.

**Table 2:** Measurement model fit indices.

Fit index	Reference value	Observed value	Interpretation
*χ^2^/*df	< 3.00	1.590	Acceptable
*IFI*	≥ 0.90 preferred	0.890	Marginal
*TLI*	≥ 0.90 preferred	0.890	Marginal
*CFI*	≥ 0.90 preferred	0.890	Marginal
*RMSEA*	< 0.08	0.041; 90% *CI* [0.037, 0.045]	Acceptable

Adaptive teaching loadings ranged from 0.298 to 0.749. Most exceeded 0.50, although AT1 (0.298) was clearly weak and AT4 (0.486) was slightly below 0.50. The composite reliability of the full 21-item adaptive-teaching construct was 0.927. A sensitivity calculation of *CR* excluding AT1 yielded 0.929, showing that overall internal consistency was not dependent on the weak item; however, a revised CFA fit after item deletion cannot be inferred from reliability calculations alone. AT1 is therefore retained in the archived model and treated as a measurement limitation rather than being deleted without re-estimation. The standardized loadings for adaptive teaching are presented in Table 3, while the SRL loadings and composite reliability results are presented in Table 4.

**Table 3:** Standardized factor loadings for adaptive teaching practices.

Construct	Item	Standardized loading
Adaptive teaching	AT1	0.298
Adaptive teaching	AT2	0.620
Adaptive teaching	AT3	0.642
Adaptive teaching	AT4	0.486
Adaptive teaching	AT5	0.601
Adaptive teaching	AT6	0.705
Adaptive teaching	AT7	0.605
Adaptive teaching	AT8	0.749
Adaptive teaching	AT9	0.506
Adaptive teaching	AT10	0.744
Adaptive teaching	AT11	0.728
Adaptive teaching	AT12	0.615
Adaptive teaching	AT13	0.606
Adaptive teaching	AT14	0.592
Adaptive teaching	AT15	0.526
Adaptive teaching	AT16	0.663
Adaptive teaching	AT17	0.576
Adaptive teaching	AT18	0.689
Adaptive teaching	AT19	0.666
Adaptive teaching	AT20	0.544
Adaptive teaching	AT21	0.679

CR for the 21-item adaptive-teaching construct = 0.927. AT1 should be interpreted cautiously because of its low loading.

**Table 4:** Standardized factor loadings and composite reliability for self-regulated learning dimensions.

Construct	Item	Loading	CR
Metacognitive self-regulation	SRL_M1	0.720	0.728
Metacognitive self-regulation	SRL_M2	0.589	
Metacognitive self-regulation	SRL_M3	0.506	
Metacognitive self-regulation	SRL_M4	0.547	
Metacognitive self-regulation	SRL_M5	0.584	
Effort regulation	SRL_E1	0.571	0.713
Effort regulation	SRL_E2	0.587	
Effort regulation	SRL_E3	0.657	
Effort regulation	SRL_E4	0.522	
Effort regulation	SRL_E5	0.540	
Time and study environment management	SRL_T1	0.627	0.807
Time and study environment management	SRL_T2	0.646	
Time and study environment management	SRL_T3	0.719	
Time and study environment management	SRL_T4	0.683	
Time and study environment management	SRL_T5	0.698	
Help seeking	SRL_H1	0.592	0.763
Help seeking	SRL_H2	0.749	
Help seeking	SRL_H3	0.656	
Help seeking	SRL_H4	0.669	

SRL was specified as a second-order construct. The four first-order dimensions loaded on the second-order factor at 0.769 for metacognitive self-regulation, 0.734 for effort regulation, 0.821 for time and study environment management, and 0.731 for help seeking. These loadings yield a second-order *CR* of 0.849. Dimension-level CR values were 0.728, 0.713, 0.807, and 0.763, respectively.

### 4.3 Structural model analysis

The structural model showed χ^2^*/*df = 2.52, *CFI* = 0.84, *TLI* = 0.83, and *RMSEA* = 0.066. The χ^2^/df and RMSEA were within commonly used ranges, whereas *CFI* and *TLI* indicated weaker incremental fit. The structural coefficients should therefore be interpreted in conjunction with this model-fit limitation rather than as evidence of a perfectly fitting model.

**Figure 1:**
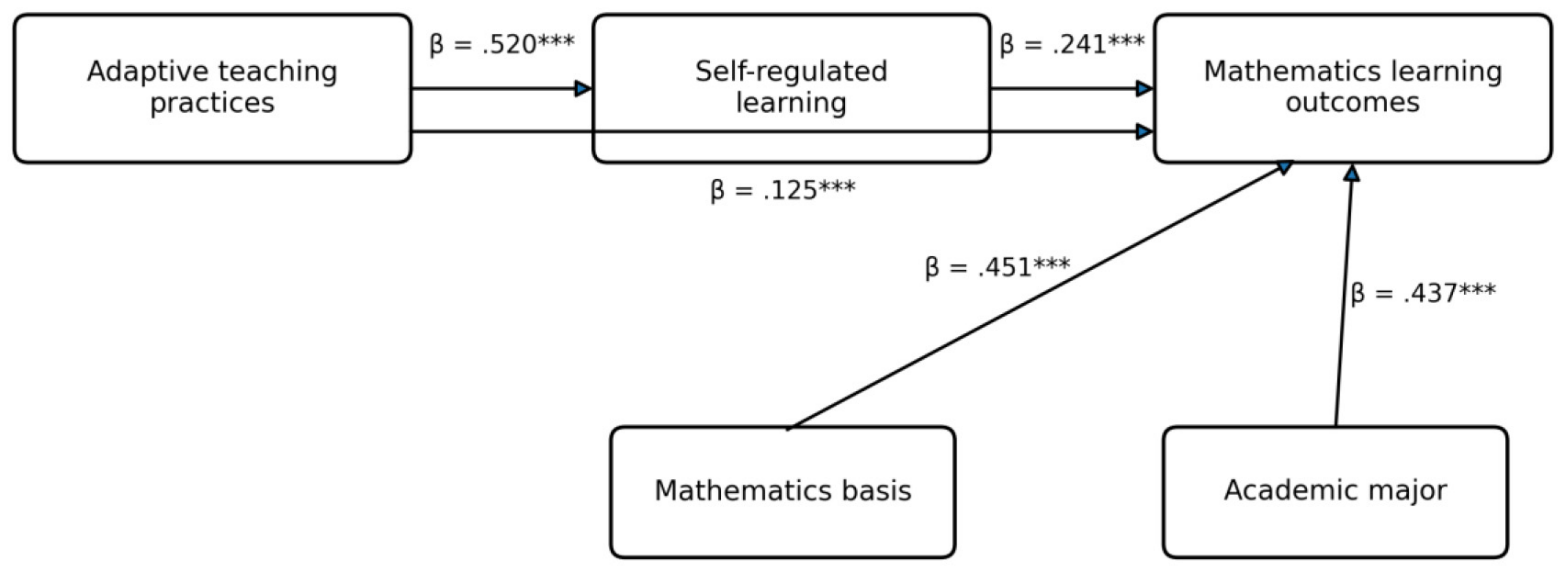
Structural equation model based on the reported standardized coefficients. ^***^p < 0.001. The indirect association through self-regulated learning = 0.125.

Adaptive teaching was positively associated with SRL (β = 0.520, *p* < 0.001), supporting H1. Adaptive teaching was also positively associated with mathematics learning outcomes (β = 0.125, *p* < 0.001), supporting H2. SRL was positively associated with mathematics learning outcomes (β = 0.241, *p* < 0.001), supporting H3. Mathematics basis (β = 0.451, *p* < 0.001) and academic major (β = 0.437, *p* < 0.001) were also significantly associated with mathematics learning outcomes. The structural associations are summarized in Table 5 and illustrated in Figure 1.

**Table 5:** Structural associations among study variables.

Hypothesis	Path	β	Result
H1	Adaptive teaching → Self-regulated learning	0.520^***^	Supported
H2	Adaptive teaching → Mathematics learning outcomes	0.125^***^	Supported
H3	Self-regulated learning → Mathematics learning outcomes	0.241^***^	Supported
Control	Mathematics basis → Mathematics learning outcomes	0.451^***^	Significant
Control	Academic major → Mathematics learning outcomes	0.437^***^	Significant

β = standardized coefficient. ^***^p < 0.001.

### 4.4 Mediation analysis

The standardized indirect association from adaptive teaching to mathematics learning outcomes through SRL was.125. This value is consistent with the product of the two relevant standardized paths (0.520 × 0.241 = 0.12532, rounded to 0.125). The archived bootstrap result from 2,000 resamples indicated that the bias-corrected confidence interval excluded zero. Because the direct association from adaptive teaching to mathematics learning outcomes remained significant after SRL was included, the pattern is consistent with partial statistical mediation. H4 was therefore supported. The cross-sectional design does not establish temporal ordering, and the result is not interpreted as evidence that adaptive teaching causally changes outcomes through SRL. The mediation results are summarized in Table 6.

**Table 6:** Mediation analysis.

Path	Standardized indirect association	Bootstrap resamples	Bias-corrected CI result	Interpretation
Adaptive teaching → SRL → Mathematics learning outcomes	0.125	2,000	Excluded zero in archived output	Partial statistical mediation

The archived manuscript retained the conclusion that the bias-corrected interval excluded zero but did not retain the numerical lower and upper bounds. Numerical bounds should not be reconstructed without the original bootstrap output.

## 5 Discussion

### 5.1 Adaptive teaching and self-regulated learning

The positive association between adaptive teaching and SRL supports H1 and is consistent with Zimmerman's view that regulation develops through interactions between learners and their learning environments. Adaptive pacing, feedback and differentiated tasks can provide information that supports forethought, performance monitoring and self-reflection. The result also accords with vocational research highlighting the importance of guidance, feedback and social support in students' regulation of learning (Pylväs et al., 2022; Mejeh and Held, 2022). Importantly, the finding does not imply that teacher adaptation substitutes for learner regulation. Rather, it suggests that supportive environmental structure and learner agency can coexist, a distinction also emphasized in recent work on system- and self-regulated learning (Herold and Seufert, 2025).

### 5.2 Adaptive teaching and mathematics learning outcomes

The positive direct association between adaptive teaching and mathematics learning outcomes supports H2. In vocational mathematics, adaptation may improve accessibility by matching task challenge to students' readiness, correcting misconceptions through responsive feedback and connecting abstract mathematics with occupational applications. This interpretation is consistent with evidence that systematic differentiation can improve learning outcomes when teaching is aligned with learner differences (Deunk et al., 2018). The effect should nevertheless be interpreted as an association because teachers may adapt instruction in response to student performance as well as influence it.

### 5.3 Self-regulated learning and mathematics learning outcomes

The positive association between SRL and mathematics learning outcomes supports H3. Within Zimmerman's framework, students who plan, monitor and regulate learning are better positioned to identify misunderstanding, persist with difficult tasks, manage time and select help-seeking strategies. These processes are especially relevant in vocational mathematics, where success depends on both conceptual understanding and the ability to apply knowledge independently. The result therefore reinforces the role of SRL as learner agency rather than as a passive response to instruction.

### 5.4 The indirect association through self-regulated learning

H4 was supported by the indirect association through SRL. The pattern is theoretically meaningful because it links an environmental instructional condition with a learner-regulation process and an educational outcome. In the broader social cognitive perspective, this is consistent with reciprocal relations among environment, personal agency and behavior. In Zimmerman's SRL framework, the result suggests that adaptive support may be related to outcomes partly through the learner's own regulatory activity. However, mediation terminology is used statistically: cross-sectional data cannot demonstrate that adaptive teaching occurred first, changed SRL, and subsequently changed mathematics outcomes. Longitudinal or experimental designs are required to test that temporal mechanism directly.

### 5.5 Theoretical and methodological contributions

The first theoretical contribution is the explicit positioning of Zimmerman's SRL theory as the primary mechanism-level framework. This clarifies learner agency in terms of forethought, performance regulation and self-reflection while retaining social cognitive theory as the broader account of person-environment interaction.

Second, the study integrates adaptive teaching and SRL within a single vocational mathematics model. This moves beyond treating instructional adaptation solely as an input and achievement solely as an output by representing SRL as a learner-level process situated between the two.

Third, the study contributes methodologically by separating measurement from structural relations through CFA and SEM and by reporting the indirect association with bootstrap inference. The revision also makes measurement weaknesses explicit rather than treating marginal fit indices and the low-loading AT1 item as unproblematic.

### 5.6 Practical implications

Vocational mathematics teachers should combine adaptive instructional support with explicit opportunities for students to regulate their learning. Differentiated tasks and flexible pacing can be paired with goal-setting prompts, self-check questions, feedback cycles and structured opportunities to seek help.

Teacher development should therefore address both adaptation and learner agency. Training can focus on using evidence of student understanding to adjust instruction while progressively transferring monitoring and strategic decision-making to students.

Curriculum designers may also embed reflective checkpoints and applied vocational problems that require students to plan, monitor and evaluate their mathematical reasoning, thereby connecting instructional responsiveness with the development of independent learning.

### 5.7 Limitations and future research

First, the cross-sectional design prevents strong causal or temporal conclusions. Longitudinal, experimental and cross-lagged designs are needed to test whether adaptive teaching precedes changes in SRL and whether those changes precede mathematics outcomes.

Second, the study relies substantially on self-report information, creating potential common-method and response biases. Future research should combine student reports with classroom observation, teacher measures, learning analytics and objective achievement indicators.

Third, the measurement model was acceptable but not optimal. Incremental fit indices were marginal and AT1 showed a low loading. Future work should refine the adaptive-teaching instrument, test item performance in independent samples and compare alternative measurement specifications.

Fourth, the study was conducted in a specific vocational education context. Replication across institutions, regions, vocational fields and age groups is needed before broad generalization.

## 6 Conclusion

This study examined adaptive teaching, SRL and mathematics learning outcomes in vocational education using a social-cognitive structural model with Zimmerman's SRL theory as the primary explanatory framework. Adaptive teaching was positively associated with SRL and mathematics learning outcomes, SRL was positively associated with mathematics learning outcomes, and the pattern included a significant indirect association through SRL.

The main contribution is conceptual rather than causal: teacher-provided adaptive support and learner self-regulation can be represented as complementary components of vocational mathematics learning. The findings suggest that instructional responsiveness may be most educationally meaningful when it is accompanied by opportunities for students to plan, monitor, regulate effort and seek help. Future longitudinal and experimental work should test the temporal mechanism more directly and refine the measurement of adaptive teaching.

## Data Availability

The raw data supporting the conclusions of this article will be made available by the authors, without undue reservation.
